# The Flail Limb Syndrome

**DOI:** 10.1002/mus.70307

**Published:** 2026-06-08

**Authors:** Mark B. Bromberg

**Affiliations:** ^1^ Department of Neurology University of Utah Salt Lake City Utah USA

**Keywords:** ALS variants, flail arm syndrome, flail leg syndrome, flail limb syndrome, motor neuron disease variants

## Abstract

The flail limb syndrome is primarily a lower motor neuron disorder that initially affects proximal arm muscles (flail arm syndrome—FAS) or distal leg muscles (flail leg syndrome—FLS). Both were recognized early on (1886 for FAS and 1918 for FLS) as somewhat distinct from classic amyotrophic lateral sclerosis (ALS). Descriptions in the literature are case series with limited information on electrophysiologic features (central and peripheral), cognitive involvement, and genetic mutations. What follows is a compilation of these features. The flail limb syndromes are rare, representing ~7%–8% of ALS. They have a higher ratio of males to females compared to classic ALS. Both are defined by predominant focal arm or leg weakness for ~2 years before progression to other regions, although there can be early and mild clinical or electrophysiologic evidence for denervation and reinnervation in other regions during the initial period. Ultimately, there is progression to respiratory failure, but at a slower rate compared to classic ALS. Upper motor neuron clinical signs are variable, but transcortical magnetic stimulation paradigms and magnetic resonance imaging tractography support upper motor neuron loss. Tests of the split hand pattern show it is rare compared to ALS. Dementia is also rare. Genetic testing supports a spectrum of ALS‐related gene mutations but at a lower frequency than with classic ALS, and no gene mutation is predominant. Diagnosis requires ~2 years of regional stability to predict the better prognosis for the flail limb syndromes.

**Abbreviations**
ALS: amyotrophic lateral sclerosis
BAD: bibrachial amyotrophic diplegia
CMAP: compound muscle action potential
CMCT: central motor conduction time
dHMN: distal hereditary motor neuropathy
EMG: electromyography
FAS: flail arm syndrome
FLS: flail leg syndrome
FTD: frontotemporal dementia
FVC: forced vital capacity
LAD: leg amyotrophic diplegia
LMN: lower motor neuron
MEP: motor evoked potential
MND: motor neuron disease
MRI: magnetic resonance imaging
NfL: neurofilament light chain
PLS: primary lateral sclerosis
PMA: progressive muscular atrophy
SICI: short interval intracortical inhibition
TDP‐43: transactive response DNA binding protein 43
UMN: upper motor neurons

## 1 Introduction

Amyotrophic lateral sclerosis (ALS) or motor neuron disease (MND) includes progressive loss of lower motor neurons (LMNs) and commonly loss of upper motor neurons (UMNs) in a range of clinical distributions that can evolve over the course of the disease. Diagnostic criteria for ALS/MND at time of initial evaluation are to ensure a uniform set of subjects for clinical studies and trials, and have been periodically updated. The first version (El Escorial criteria) is based on the relative distribution of UMN and LMN findings along rostro‐caudal body regions (bulbar, cervical, thoracic, lumbosacral) leading to “definite,” “probable,” “possible” and “suspected” ALS [1]. A gray diagnostic pattern is only LMN involvement in two regions (progressive muscular atrophy—PMA) and is “suspected” ALS, but subsequent revisions (Airlie House and Awaji) removed “suspected” ALS and hence PMA from fulfilling criteria [2, 3]. The most recent version (Gold Coast criteria) simplifies diagnoses to “ALS” or “not ALS,” based on experience that survival curves are similar for the range of “definite” to “suspected” ALS [4]. Accordingly, progressive LMN dysfunction in at least two body regions is considered to be ALS, and includes PMA. The diagnosis of ALS is excluded when there is only UMN involvement (primary lateral sclerosis—PLS).

The flail limb syndromes are variants of ALS/MND with distributions of progressive LMN involvement that predominately affect either arms (flail arm syndrome—FAS) or legs (flail leg syndrome—FLS) for a significant portion of time, and which may or may not include UMN findings. The two patterns are distinct and were recognized early on. The first description of progressive proximal arm weakness in 1886 by Vulpain became known as the Vulpain‐Bernhardt form [5], and Gowers in his textbook [6] included an illustration of a patient with arms hanging limp. Subsequent authors have included photographs of similarly affected patients [5]. The first contemporary review in 1998 offered the term “flail arm syndrome,” [7] and other names include hanging‐arm syndrome, suspended form, orangutan sign, dead arm sign, man‐in‐a‐barrel, brachial amyotrophic diplegia, bibrachial palsy, rizomelic atrophy [8]. Progressive distal leg weakness described in 1918 by Patrikios as pseudopolyneuritic or peroneal variant [9] became known as the Marie‐Patrikios form and later “flail leg syndrome,” [10] and another term is “leg amyotrophic diplegia” (LAD) [11].

The clinical features of flail limb syndromes have been described in case series and small reports and specific aspects and features reported, but a contemporary review that includes clinical, nonmotor, pathologic, electrophysiologic, and genetic features as well as unusual features is warranted now that patients with flail limb syndromes can be classified as ALS by Gold Coast criteria.

## 2 Methods

PubMed and Google Scholar databases were searched using terms “fail limb syndrome,” “flail arm syndrome” and “flail leg syndrome,” and references from extracted articles were also reviewed. Single case reports and small case series were not included unless they presented pathology or genetic test results.

### 2.1 Operational Definitions

Clinical definitions for FAS and FLS were formalized by Wijesekera et al. and include [10]:

- *Overall*: Progressive weakness and muscle wasting.
- *FAS*: Predominant proximal upper limb involvement for at least 12 months after symptom onset.
  − Excludes distal limb wasting and weakness without proximal involvement and functionally significant weakness and wasting in legs and bulbar region within 12 months of onset in upper limbs.
- *FLS*: Predominant distal lower limb weakness for at least 12 months after symptom onset.
  − Excludes wasting or weakness beginning proximally in legs, functionally significant weakness and wasting in upper limbs, bulbar region or respiratory musculature within 12 months after onset in upper limbs.
- Patients may have pathologic stretch reflexes in the respective region.
- *Exclusions*: Hypertonia in upper and lower limbs and ankle clonus.

Case series for FAS before 2009 are not strict on duration of clinical stability in the arms (but measured in years) [7, 12] and the series by Vucic et al. uses 24 months [13], while case series after 2009 use the clinical definition of Wijesekera et al. [10]. Early case series of FLS use more restrictive clinical features with weakness limited to legs and absence of clinical upper motor neuron findings for ≥ 2 years, and offer the term LAD [11], while later series used criteria of Wijesekera et al. [10].

### 2.2 Tabulated Data

Clinical data are collated and tabulated in Tables 1, 2, 3, 4 from case series with reasonable sample sizes. Most case series describe patient features at the time of diagnosis with varying details about changes over time.

**TABLE 1:** Demographic features of patients with flail arm syndrome compared to classic amyotrophic lateral sclerosis (ALS), from references.

References	Amyotrophic lateral sclerosis (Bulbar + Limb)				Flail arm syndrome						
	Number	M:F	Median onset (years)	Median/mean survival (months)	Number	% of ALS	M:F	Median onset (years)	Median/mean survival (months)	UMN involvement (%)	Dementia (%)
Hu [7]	395	1.5:1	55	39	39	10	9:1	58	57	77	
Katz [14]					10	2	1.5:1	53.3		10	
Couratier [12]	357	1.35:1	66.3	32.8	20	6	4:1	59.8	49.5	50	
Vucic [13]		1.5:1	58.3	15.8	11		10:1	60.3	61.5	40	
Wijesekera London [10]	1188	1.5:1	59.6	35	135	11	4:1	57.3	61	NA	
Wijesekera Australia [10]	432	1.1:1	63.8	31	22	5.1	10:1	61.2	66	NA	
Chio [15]	860	1.32:1	65.8	24.3	74	9	4:1	63.3	48		1.4%
Yoon [16]	578				18	3	5:1	59		44	
Hübers [17]	146	1.9:1	59.4	33	42	NA	3.8:1		53	NA	
Chen [18]	1935				117	6	8.75:1	55	88.6		
Schito [19]	1207	1.13:1	65.7	38	117	10	2.44:1	62.3	91	NA	6.8%
Xu [20]					130	9	5:1	52.2		NA	
Gromicho [21]					56	8	3:1	59.9	109.5	46.4	1.9%
Shen [22]	1305	0.95:1	54	29	109	7	2.03:1	52.6	56		
Summaries	Number 8403	Average 1.36:1	Average 60.9	Average 30.88	Number 900	Average 7.18	Average 5.18:1	Average 58.02	Average 67.37	Average 44.57	Average 3.4%

Abbreviations: ALS, amyotrophic lateral sclerosis; M:F, male to female ratio; No: number of subjects; UMN, pathologic reflexes.

**TABLE 2:** Clinical features of patients with flail arm syndrome, from references.

	Clinical features						El Escorial, Awaji criteria					
	No	Bulbar involvement	Leg involvement	EDX beyond arms	Clinical UMN	TMS	Definite	Probable	Laboratory supported	Possible	Suspected	Unclassified
Hu [7]	39	56% FU			70%		Most			Minority	Minority	
Katz [14]	10	0		20% Subclinical	10%		0%	0%		0%	90%	
Couratier [12]	20	35%			70%							
Vucic [13]	11	27%	18%		0%	100%						
Wijesekera London [10]	135	< 46%	< 46%				1.5%	20%	31.9%	21.5%	25.2%	
Yoon [16]	18			> 80%	53%				40%	17%		
Hübers [17]	42			66.7%								
Chen [18]	117						10%	11%	15%	35%		38%
Schito [19]	117						10.3%	18.8%	25.6%	28.2%		17.1%
Xu [20]	130				97%	97%						
Gromicho [21]	56			> 85%	46.4%							
Summary	695						Average 5.5%	Average 12.5%	Average 28.1%	Average 20.3%	Average 57.6%	Average 27.6%

Abbreviations: EDX, electrodiagnostic abnormalities (nerve conduction and EMG); No, number of subjects; TMS, transcranial magnetic stimulation abnormalities; UMN, pathologic reflexes.

**TABLE 3:** Demographic features of patients with flail leg syndrome compared to classic amyotrophic lateral sclerosis (ALS), from references.

References	Amyotrophic lateral sclerosis (Bulbar + Limb)				Flail leg syndrome						
	Number	M:F	Median onset (years)	Median/mean survival (months)	Number	% of ALS	M:F	Median onset (years)	Median/mean survival (months)	UMN involvement (%)	Dementia (%)
Cappellari [23]					8		3:1	56		20	
Wijesekera London [10]	927	1.5:1	60.3	30.1	75	8	0.92:1	55.0	69		
Wijesekera Australia [10]	432	2.1:1	63.8	29	12	3	5:1	59	71		
Chio [15]	860	1.32:1	65.8	24.3	173	13	1.03:1	65	36		4.0%
Dimachkie UofK [11]					8		3:1	58	87	0	
Dimachkie US [11]					16	2.5	15:1	57	87	0	
Menon [24]	119	1.45		27.5	18	12.9	0.8:1		38	78	
Shito [19]	1612	1.13:1	65.7	33	148	10.9	1.47:1	63.7	62		4.8%
Zhang [25]					29		1.4:1	49.6	80	36.2	
Summaries	Number 3950	Average 1.55	Average 63.9	Average 28.8	Number 488	Average 8.4	Average 3.52	Average 58.91	Average 66.3	Average 26.84	Average 4.4%

Abbreviations: ALS, amyotrophic lateral sclerosis; M:F, male to female ratio; No, number of subjects; UMN, pathologic reflexes.

**TABLE 4:** Clinical features of patients with flail leg syndrome, from references.

	Clinical features						El Escorial, Awaji criteria					
	Number	Bulbar involvement	Arm involvement	EDX beyond arms	Clinical UMN	TMS	Definite	Probable	Laboratory supported	Possible	Suspected	Unclassified
Cappellari (2008) [24]	8	0%	50%	88%	63%	89%						
Wijesekera London (2009) [9]	75						0%	22.7%	17.3%	26.7%	33.0%	
Dimachkie UofK (2013) [10]	8	0% (definition)	0% (definition)	0% (definition)	0% (definition)		0%	0%	0%	0%	0%	
Dimachkie US (2013) [10]	16		31% > 2 years									
Menon (2016) [26]	18		56%		78%			17%		50%	33%	
Schito (2020) [27]	148						14.2%	23.6%	18.2%	25.1%		18.9%
Zhang (2022) [28]	29				37.9%							
Summary	302						Average 4.3%	Average 15.8%	Average 11.8%	Average 25.5%	Average 22.0%	Average 18.9%

Abbreviations: EDX, electrodiagnostic abnormalities (nerve conduction and EMG); TMS, transcranial magnetic stimulation abnormalities; UMN, pathologic reflexes.

## 3 Flail Arm Syndrome

### 3.1 Clinical Features

Data in Table 1 are from case series of patients who fulfill the initial period of 12–24 months of predominant proximal arm weakness to support the diagnosis of FAS. Some series include comparisons of features between FAS and classic ALS (combining limb and bulbar onset). There are consistent findings across series for FAS compared to classic ALS, with greater numbers of affected males and longer mean/median survival FAS compared to ALS, although both differences within a series rarely reach statistical significance. Descriptions of distributions of weakness within the upper extremities at presentation vary among series, and at onset small to moderate percentages of patients have initial distal upper extremity weakness (intrinsic hand muscles) with uniform progression to proximal > distal weakness to fulfill the diagnostic distribution. Asymmetry is common at onset, progressing to bilateral involvement over 2 plus years. Table 2 shows the El Escorial and Awaji diagnostic classifications, with most patients fulfilling “probable” to “suspected” ALS or unclassified, and rarely “definite” ALS [1, 3]. Patients can live beyond the observation period (> 2 years) or are lost to follow up, leading to variable documentation of progression of weakness to rostral and caudal regions, respiratory insufficiency and use of noninvasive ventilation, and death. Eight patients with brachial amyotrophic diplegia (BAD) are unusual with preserved ability to ambulate independently and with no respiratory insufficiency 3–10 years from symptoms onset [14].

Despite predominant involvement of muscles innervated by 5th and 6th cervical roots, the diaphragm is involved to a lesser degree early on (~2 years of onset) than in classic ALS, leading to the above survival advantage. A study of 11 patients with FAS showed a 0.29% points per month average percent change (decline) in forced vital capacity (FVC) compared to a 3.8% per month change in 16 patients with classic ALS [29]. Another comparison indicated a median time for need for non‐invasive ventilation at 61.6 months from symptoms onset for FAS and 24.4 months for classic ALS patients [10]. In contrast, five Japanese patients with proximal arm weakness developed respiratory failure over a median of 14 months (5–20 months), but the authors argue that these patients are within the overall prolonged range of survival in the literature of patients with FAS (6–109 months) and that some considered to have FAS will survive less than the 24 months [30]. Another report compares 22 patients with FAS to 329 patients with ALS at > 12 month into the disease course, and significantly fewer patients with FAS have an FVC < 80% compared to ALS patients (18% vs. 45%) [31]. A study of 56 patients with FAS who had UMN findings were shown to have significantly shorter survival (69.5 months) than those without UMN findings (152.6 months) [21]. For patients considered to have BAD, two reports of 2 and 10 patients, respectively, indicate respiratory failure > 5 years from symptom onset and with no significant involvement of lower extremity muscles [14, 32].

The presence of UMN findings (pathologically brisk tendon reflexes and Hoffmann signs) is not always commented upon, but an average of ~45% of patients have pathologic reflexes, more frequent in lower than upper limbs (Table 1) [7, 12, 16, 20, 21].

Case reports of FAS in unique settings include three Spanish former football (soccer) professional and semi‐professional players with onset ages 32.5, 32, and 45.6 years [27].

There is little information on the frequency of dementia, but reports mention “comorbid dementia” [21], “dementia” [19], and “frontotemporal dementia” [15], with an average frequency of 3.4% (Table 1).

### 3.2 Electrophysiology Features

Routine peripheral electrodiagnostic findings are reported in varying detail. Nerve conduction studies are within normal limits except for reduced compound muscle action potential (CMAP) amplitudes when stimulating upper extremity nerves with no evidence for conduction block, although one study described changes in median sensory nerves in several patients [33]. Electromyography (EMG) demonstrates active denervation (positive waves/fibrillation potentials) and chronic reinnervation (decreased recruitment, enlarged and complex motor unit potentials). At time of diagnosis, these findings are observed mainly in arm muscles and may also be found in bulbar and leg muscles to minor degrees (Table 3) [1, 16, 17].

The split hand pattern has been assessed using an abbreviated index (abductor digiti minimi CMAP amplitude divided by the abductor pollicis brevis CMAP amplitude) comparing six patients with FAS and 18 control subjects and showed no differences in mean index values (1.24 vs. 1.22), while the index was significantly higher in 41 ALS patients (2.36) [34]. A second study showed no difference between 11 subjects with FAS (0.9) and 40 controls (1.0), but 36 with ALS had a higher ratio (2.65) [35]. A third study (reversing the numerator and denominator in the index) found no difference in the index values between nine patients with FAS and 20 controls (1.11 vs. 1.05), while the index was lower in 43 patients with upper limb onset ALS (0.56) [36].

Transcranial magnetic stimulation of the motor cortex to assess cortical excitability and corticospinal (UMN) integrity has been assessed. Eight subjects with FAS (none with UMN findings) compared to 26 subjects with classic ALS and with control subjects showed similar central motor conduction times for the three groups (FAS 5.9 ms, ALS 5.1 ms, control 5.1 ms) [13]. Resting motor thresholds were similar between FAS (53.4%) and ALS (56.5%), but reduced compared to control subjects (60.7%). Motor evoked potential amplitudes (as percentage of CMAP amplitude) were increased in FAS and classic ALS compared to control subjects. Similarly, short interval intracortical inhibition (SICI) was significantly reduced in FAS and classic ALS compared to control subjects. Intracortical facilitation was increased in FAS compared to controls. These data support cortical hyperexcitability in FAS despite variability of clinical UMN findings. Another study included six subjects with FAS (none with UMN findings), 41 with ALS and 18 control subjects. Central motor conduction times were similar across the three groups (FAS 7.9 ms, ALS 10.0 ms, control 8.4 ms) [34]. Resting motor evoked potential amplitudes between FAS and control subjects were similar but lower than in ALS subjects (values not provided). Based on finding no increase in resting motor thresholds in subjects with FAS compared to control subjects, the authors concluded that there was no increased central hyperexcitability in FAS compared to control subjects, but they do not speculate as to why their results differed from those in the Vucic et al. study [13].

### 3.3 Pathologic Features

Findings are based on few cases. A case clinically consistent with FAS remained ambulatory for 8 years with progression to bulbar muscle weakness and death at 10 years showed severe LMN loss in the cervical but not lumbar spinal cord, the presence of Bunina bodies but no corticospinal tract degeneration in the cervical cord, and unique vacuoles in intact lumbar motor neurons [37]. Two cases with predominant arm weakness and denervation in all limbs and with survival of 2.3 and 11 years, respectively, showed LMN loss, no Bunina bodies, but TDP‐43 inclusions in astroglia [26].

### 3.4 Genetic Features

Older case series excluded patients with familial ALS and others report no family history among FAS patients, while recent series include screening panels for common ALS gene mutations for a sample of patients. (Table 5, Upper). Anecdotal comments include four patients with FAS with a family history of ALS; two patients had *TARDBP* mutations, but no other affected family members had the FAS phenotype, and two patients had no mutations on gene screening [21].

**TABLE 5:** Gene mutations associated with flail arm (upper) and flail leg (lower) syndromes.

Gene mutations flail arm syndrome									
	*SOD1*	*C9orf72*	*TARDP*	*FUS*	*hnRNPA1*	*SPG7*	*OPTN*	*CRN*	*PEN1*
Gromicho [21]		1/32	c.1154G>T, p.(TRP385Leu)						
Schito [19]	1/39^a^						0/39	0/39	
Liu [38]					c.862/1018C>T, p.P288S/P340S				
Han [39]					c.862/1018C>T, p.P288S/P340S	*c.1457G>A* *c.1529C>T*			
Osmanovic [40]						c.1457G>A c.1529C>T			
Gene mutations flail leg syndrome									
Schito [19]	5/65^a^	1/65^a^	1/65^a^	0/65			0/65	0/65	
Zapalska [41]	L144S (c.434 T>C, p.Leu145Ser)								
Osmanovic [40]						c.1045G>A			
Taieb [42]				R521C					
Zou [43]									c.353G>T (p.G118V)
Ricci [44]	c.355G>Ap.V119M								

^a^ Mutation not specified.

## 4 Flail Leg Syndrome

### 4.1 Clinical Features

Definitions of FLS vary among case series, but all include focal involvement of distal leg muscles for ≥ 12 months (commonly > 2 years), while those with LAD require clinical and EMG abnormalities to be confined to leg muscles for > 2 years (Table 3) [11]. The sex ratio varied among case series with higher average ratios favoring males compared to classic ALS, but differences were not significant within studies. The average age of symptom onset for FLS is younger than for classic ALS, but not statistically significant within studies. Onset may be in one leg with progression of weakness from distal (initial foot drop is common) to proximal muscles and to the other leg, or initial proximal weakness, but there is overall predominant distal leg weakness. Spread to upper limbs and bulbar muscles occurs eventually (excluding LAD) over > 18 months. All series include survival data for the period of observation but ultimate progression to respiratory insufficiency and need for non‐invasive ventilation is not always described. Survival is very prolonged compared to classic ALS. Classification by El Escorial or Awaji criteria is weighted to “laboratory supported” and unclassified (Table 4).

UMN involvement is an exclusion for LAD [10]. For other series it can be present at onset or over time, more common with progression to arm muscles [24], and not commonly reported in other series; overall it is very low, and it is pointed out that reflexes may be reduced or not present in the legs due to LMN loss.

Dementia is rarely commented upon and referred to as “Frontotemporal dementia” [19] and “dementia,” [15] and the average is 4.3% (4.0%–4.8%) (Table 3).

### 4.2 Electrophysiology Features

Peripheral electrophysiology shows nerve conduction studies within normal limits in arms, and in the legs sural responses are normal but CMAPs are reduced in amplitude or absent, with no evidence for conduction block [11, 23]. EMG studies show abnormal spontaneous activity and neurogenic motor unit potentials in leg muscles, and fasciculation potentials in arm muscles, but motor unit potentials are normal in cervical and bulbar muscles.

Central electrophysiology studies include 18 subjects (using the abductor pollicis brevis muscle) and show SICI to be significantly reduced in FLS (7.2 ms) compared to control subjects (13.2 ms) and longer compared to classic ALS patients (value not provided), but significantly reduced in FLS with UMN clinical findings compared to those without [24]. Motor evoked potentials are higher in FLS (29.2 mV) compared to control subjects (18.9 mV) and similar to those with classic ALS. CMCT is significantly longer in FLS patients compared to controls (6.7 ms vs. 5.6 ms) but similar to classic ALS (6.5 ms). In another study of eight patients, one with reduced MEP and delayed CMCT values and three with borderline CMCT values, reduced or absent MEG and delayed CMCT were found in 93.7% recorded from leg muscles [23].

### 4.3 Pathologic Features

Findings are based on single cases, and with uncertainty in the use of the term “pseudoneuritic” being restricted clinically to legs consistent with criteria for FLS, although there can be progression to other regions over the time to postmortem examination. Among three cases of progressive distal lower extremity weakness, two were termed pseudoneuritic but progressed from unilateral leg onset to arm and bulbar weakness and death within 25 and 39 months, while one case with the slow progression of FLS showed diffuse LMN loss throughout the spinal cord with no Bunina bodies or TDP‐43 positive staining, and there was no corticospinal tract degeneration [45].

### 4.4 Genetic Features

Mutations associated with FLS are in Table 5 Lower.

## 5 Features Related to FAS and FLS

Several features are common to both FAS and FLS but with limited data.

### 5.1 Imaging Findings

Routine brain magnetic resonance imaging (MRI) shows no specific findings. Diffusion tensor imaging assessing fractional anisotrophy showed change in corticospinal tracts in one subject with FAS and three with FLS [46]. A larger study of 43 patients with FAS compared to 43 patients with classic ALS and 40 control subjects showed that patients with FAS display involvement of the corticospinal tracts identical to those with ALS [47]. Routine cervical spine MRI generally shows no specific findings. A case description of a 22 year old male with 2 years of weakness involving bilateral upper extremities had linear T2 intramedullary hyperintensities in transverse section described as an “owl's eye sign” [48]. Three males with progressive arm atrophic weakness not clearly consistent with FAS had “snake eye signs” on transverse cervical MRI sections and hyperintensity signals along several segments (2–6 segments) [49]. Thus, T2 hyperintensities in the anterior cervical spinal cord (snake or owl eyes) are not specific for FAS, and are described mostly in case reports associated with cervical spondylosis, cord infarction, Hirayama disease and cord ischemia. A series of 29 subjects with snake eye signs, predominantly male (86.2%) and young (mean age 37.3 ± 14,4 years) and with no clinical UMN loss, were followed for an average of 9.5 years with 10% diagnosed with Hirayama disease, 28% cervical stenosis and 62% with no etiology [50].

### 5.2 Neurofilament Light Chain

Cerebrospinal fluid neurofilament light chain (NfL) levels were significantly higher in 94 subjects with classic ALS compared to control subjects, and lower in subjects with predominantly LMN distributions (six patients with FAS, five with FLS, and nine with PMA) than those with ALS [28]. Serum NfL levels were significantly higher in 74 subjects with classic ALS compared to 15 with FAS or FLS, with mean values about half those with ALS [25]. A large study found that patients with bulbar onset ALS had the highest serum NfL levels compared to limb onset ALS, while those with flail limb syndromes were lower than all ALS, and FLS values somewhat higher than FAS [51].

## 6 Discussion

The relative distributions of LMN loss are distinct between the two flail limb patterns, with proximal > distal loss in FAS and distal > proximal loss in FLS, with no speculation in the literature as to factors accounting for the differences. There is a sex difference for the two phenotypes with more affected males compared to classic ALS, also with no solid speculations. For both distributions, weakness eventually progresses rostrally and caudally to include death from respiratory failure (although case series are time limited to determine ultimate progression). The median/mean survival is longer for FAS and FLS than for classic ALS, and is markedly prolonged in the LAD form. Both syndromes have percentages of UMN involvement that vary across studies (0%–78%), as with classic ALS, with support for involvement from central electrophysiology and tensor MRI imaging. Dementia occurs but rates are markedly lower than in classic ALS. Distinctions among types of dementias are not always clear but case series reporting dementias are from 2011 onward, the time period for the recognition of and development of clinical FTD testing. Pathology reports are few and clinical features represent end stage progression, but findings for both patterns overlap with those in classic ALS. Gene mutations seem to be rare and overlap with those in ALS, and no unique gene mutation is linked to either flail limb disorder. An interesting observation is that patients with SOD1 D91A mutations can have clinical features similar to those described by Patrikios (including painful pre‐paretic symptoms) and at autopsy have pyramidal and posterior column fiber loss [52]. Overall, FAS and FLS can be considered clinically unique variants of ALS with initial LMN loss affecting segmental body regions and with longer survival.

The primary differentiation for FAS early in the clinical course is upper limb onset ALS, and two case series compare features between the two clinical entities. A study compared 56 Chinese patients with upper limb onset ALS to 18 with FAS. The male: female ratio in upper limb onset ALS (1.4:1) was similar to that of classic ALS (1.8:1 from Table 1), while the sex ratio of FAS was higher (5:1) [16]. Another study compared 767 Chinese patients with upper limb onset ALS to 117 with FAS and also found a lower sex ratio in upper limb onset ALS (1.81:1) compared to FAS (8.75:1), and median survival shorter for ALS (1887 days) than for FAS (2694 days) [18]. There are no direct comparisons between FLS and classic ALS with lower limb onset; however, a review of the clinical course of 144 patients with leg onset (with no separation of those with FLS pattern) indicated that most experienced single limb onset of weakness with progression to the contralateral leg in 76% and to the ipsilateral arm in 24%. The median time to progression to the contralateral leg was 21 months [53]. The sex ratio was 1:1 with no effect on progression or survival. Thus, there is likely a difference between leg onset ALS and FLS.

The diagnosis of flail limb syndromes is aided by the clinical Wijesekera et al. criteria [10] (Section 2.1) with an emphasis on clinical focality for ~2 years before progression to other regions. Nerve conduction studies exclude conduction block and EMG studies show prominent evidence for acute and chronic denervation in the primary region. Few reports on flail limb syndromes include alternative diagnoses to consider. For FAS, proximal weakness leading to the hanging arm posture is unique, but the rare condition of bibrachial amyotrophy due to intraspinal fluid collection can lead to a similar pattern of proximal arm weakness [54]. Multifocal motor neuropathy should be excluded, but unlike FAS, it has a mononeuropathy pattern often with distal weakness. FLS and unilateral multifocal motor neuropathy can have overlapping features, and a report of two patients with distal asymmetric leg weakness after 5 and 2 years, respectively, led to further investigations and the diagnosis of multifocal motor neuropathy with very proximal conduction block that responded to intravenous immune globulin treatment [55]. Bilateral distal leg weakness early in the course raises the possibility of a distal hereditary motor neuropathy (dHMN) [56]. Pathogenic gene variants in dHMN can be autosomal dominant or recessive, although sporadic cases are also reported; most present at a younger age than flail limb syndromes but can present in adulthood.

Fulfillment of Gold Coast criteria [4] for ALS by the flail limb syndromes at time of diagnosis can be problematic as the main clinical manifestation is prominent LMN loss in a single rostrocaudal region, and most patients do not meet criteria at this time point. For FAS, patients can have some electrophysiologic evidence for denervation in cervical and to a mild degree in lumbosacral regions early on, and many have or develop pathologic reflexes over 2 years, and thus some can meet criteria within 2 years. For FLS, weakness seems to be restricted to the legs for many years with some patients having only mild evidence for LMN loss (fasciculation potentials) in cervical region muscles. Detection of UMN findings in the legs may be blunted by LMN loss. For both forms, cortical electrophysiologic testing and special MRI sequences indicate UMN loss in most tested patients, but routine central electrophysiologic testing is not practical. Ultimately there is spread to the bulbar region and respiratory failure, as in ALS, and most patients will fulfill criteria for ALS, but not at the initial evaluation. With a more favorable prognosis of FAS and FLS than for classic ALS, it would be desirable to predict the expected clinical trajectory early in the disease course, but it requires at least 2 years of observation, and early prediction is not reliable.

## Author Contributions

**Mark B. Bromberg:** conceptualization, methodology, writing – original draft, writing – review and editing, investigation, formal analysis, data curation.

## Funding

The author has nothing to report.

## Ethics Statement

I confirm that I have read the journal's position on issues involved in ethical publication and affirm that this report is consistent with those guidelines.

## Consent

The author has nothing to report.

## Conflicts of Interest

Mark Bromberg has received no support that is a direct conflict; total support includes: Accordant Health Care: Medical Advisor, Oxford University Press: Royalties, Cambridge University Press: Royalties, UpToDate: Royalties, and AMGEN: Data Safety Monitoring.

## Data Availability

The data that support the findings of this study are available from the corresponding author upon reasonable request.
